# Cognitive tasks during walking affect cerebral blood flow signal features in middle cerebral arteries and their correlation to gait characteristics

**DOI:** 10.1186/s12993-015-0073-9

**Published:** 2015-09-26

**Authors:** Arthur Gatouillat, Héloïse Bleton, Jessie VanSwearingen, Subashan Perera, Scott Thompson, Traci Smith, Ervin Sejdić

**Affiliations:** Department of Electrical and Computer Engineering, Swanson School of Engineering, University of Pittsburgh, Pittsburgh, PA USA; Department of Physical Therapy, School of Health and Rehabilitation Sciences, University of Pittsburgh, Pittsburgh, PA USA; Division of Geriatric Medicine, Department of Medicine, University of Pittsburgh, Pittsburgh, PA USA

**Keywords:** Transcranial Doppler, Gait accelerometry, Cerebral blood flow velocity, Walking, Signal features

## Abstract

Gait is a complex process involving both cognitive and sensory ability and is strongly impacted by the environment. In this paper, we propose to study of the impact of a cognitive task during gait on the cerebral blood flow velocity, the blood flow signal features and the correlation of gait and blood flow features through a dual task methodology. Both cerebral blood flow velocity and gait characteristics of eleven participants with no history of brain or gait conditions were recorded using transcranial Doppler on mid-cerebral artery while on a treadmill. The cognitive task was induced by a backward counting starting from 10,000 with decrement of 7. Central blood flow velocity raw and envelope features were extracted in both time, frequency and time-scale domain; information-theoretic metrics were also extracted and statistical significances were inspected. A similar feature extraction was performed on the stride interval signal. Statistical differences between the cognitive and baseline trials, between the left and right mid-cerebral arteries signals and the impact of the antropometric variables where studied using linear mixed models. No statistical differences were found between the left and right mid-cerebral arteries flows or the baseline and cognitive state gait features, while statistical differences for specific features were measured between cognitive and baseline states. These statistical differences found between the baseline and cognitive states show that cognitive process has an impact on the cerebral activity during walking. The state was found to have an impact on the correlation between the gait and blood flow features.

## Background

Walking is a complex sensory-cognitive interaction which has various demands depending on the environment [1–3]. Initially, cognition and motor control of gait have been believed to be two completely autonomous processes, with walking regarded as an automatic motor function, independent of any cognitive tasks [4, 5]. Walking was thought to be automatic as it is generated by spinal cord oscillating circuits, or at best, locomotor centers in the brain stem but without cortical input under usual conditions. If that were true, there should be little if any interference of cognitive functioning with walking—but recent research shows that a cognitive load has an effect on gait [6–8]. In fact among people with pathological conditions, the dual-task methodology was mainly used to observe the effect of concurrent stimuli while checking the gait state [9]. This technique may underline a cognitive-motor interference that indicates a conflict between concurrent tasks (i.e. a motor and a cognitive challenges), as there may be a deterioration of one or both of the tasks [9, 10].

In general, a dual task (i.e. a cognitive stimulus/walking) causes concurrent demands for attention/cognitive resources. Hence, it generates a cognitive-motor interference that implies tripping, falling, physical instability and/or a decline in performance [2, 4, 11]. These falls occur mainly in elderly subjects and have a strong impact on the health of elderly subjects [6]. These events also have an impact on the health costs [12] and the risk of falling is associated with gait disruptions [13]. Prior studies have illustrated that individuals with neurodegenerative disorders, those recovering from stroke or elderly adults, are more prone to the effects of cognitive load on gait [7, 9, 14]. On the other hand, just a few investigations have investigated the cerebral repercussions of a dual-task in the case of able-bodied young participants [15]. Moreover, there has been limited research on the effects of cognitive load on the cerebrovascular system during gait.

Thus, there is a growing interest in clarifying the correlation between motor control and cognition. Brain imaging methods have revealed activation of various cerebral regions associated with higher cognitive functions during walking (i.e. the dorsolateral prefrontal cortex and anterior cingulate cortex [16–19]).

However, because of the lack of portability of these devices and the fact that they require the subject to lie down during acquisition, the gait is usually modeled as a series of feet tapping in the fMRI case [20, 21] and it is studied by the injection of a radio-tracer, followed by the subject’s walk, and a scan for the PET/SPECT case [22, 23]. These devices, because of their lack of portability and the strong constraints associated with their use, are not optimal for performing a real time study of the brain during walking. These drawbacks led us to chose CBFV recordings using transcranial Doppler to assess brain function during walking.

Since it has been proven that CBFV modifications and neural activity can be correlated [24–28], we use transcranial Doppler recordings to monitor the hemodynamic activities of the main cerebral arteries [29] in order to study a motor-cognition interaction. This non-invasive ultrasound diagnostic tool first introduced by Aaslid et al. measures the cerebral blood flow velocities (CBFV) [30]. Readings are taken with ultrasonic transducers, placed bilaterally in the transtemporal window of the skull of one participant, which allows the monitoring of the left and right side of the circle of Willis’ cerebral arteries [31–34]. Most studies focused on activities of the middle cerebral artery (MCA) given that the MCA carries more than 80 % of blood to the brain [35]. Moreover, previous papers highlighted brain perfusion changes during neural cognitive challenges [36–38]. In concern to physical performance, global cerebral blood flow (CBF) appears to be increased, unchanged or decreased during stimuli [39–41]. Nevertheless, a regional increase of CBF was noticed during physical exercise [42].

The current study was focused on studying the associations between the cerebrovascular system and gait and understanding the repercussions of cognitive load during walking on the system. Specially, we examined CBFV signals in MCA. Our major contributions are the statistical differences found in the central blood flow velocity signals between the baseline and cognitive states during walking. We examined both raw signals and the envelope signals, which are maximum peak velocity outcomes. In addition, baseline is actually walking—and on a treadmill which is a dedicated stepping pattern facilitator, walking could be even more automatic than usual—yet the blood flow changed with added cognitive processing; and the changes in CBF consistency mirrored in part the changes in gait consistency—at least for the performance of some—illustrating the potential interference of cognitive functioning and walking.

## Methods

### Data acquisition

For this preliminary data collection, eleven participants were recruited (4 females and 7 males, ages ranging from 19 to 23 years). None of these subjects had history of concussion, heart murmurs, migraines, strokes or other brain and gait conditions. Participants were asked to signed the University of Pittsburgh Institutional Review Board approved consent form and the procedure was explained prior to the beginning of the experiment and data collection.

The participants were briefed to walk at a pace of 2 mph on the Noraxon MR3 treadmill and to remain thought-free during this 6 min baseline testing period. Upon the completion of this trial, the participants were asked to count backwards from 10,000 in decrements of 7 while walking for 6 min for another trial referred as cognitive. Approximately 1 week later, each participant repeated the experiment.

To observe stride time, Noraxon SciFit treadmill using capacitive sensor technology to analyze individual foot pressure (ranging from 1 to $120~\,N /cm ^2$) was used at a sampling frequency of 100 Hz. The treadmill data was recorded and extracted using the manufacturer’s software.

A SONARA TCD System (Carefusion, San Diego, CA, USA) was used to measure blood-flow velocity in the mid-cerebral artery. Two 2 MHz transducers were used to gather simultaneous bilateral CBFV acquisitions from the left-MCA and right-MCA. The transducer were planted on transtemporal windows [43] to reach the MCA blood-flows. The position, angle and insonation depth of transducers were adjusted in order to get the correct MCA signal [34, 44]. Once these adjustments were performed, the transducers were fixed with a headset on both sides of the subject’s head. The data was extracted as audio files sampled at $44.1\,~kHz$, representing the cerebral blood flow from the R-MCA and L-MCA. These signals were resampled at 8820 Hz (factor 5) to increase the feature extraction’s speed. The downsampled signals, referred as raw signals, are composed of multiple sinusoidal components due to the parabolic speed CBFV distribution [37]. In this study, these raw CBFV along with the envelope of the CBFV were collected. The raw signals are composed of the various velocities of blood particles in cerebral arteries and envelope signals constitute the maximal Doppler shift [37, 45].

Furthermore, the end-tidal carbon dioxide $ETCO _2$ (BCI Capnocheck Sleep Capnograph, Smiths Medical, Waukesha, Wisconsin, USA) was monitored along with respiration rate, electrocardiogram, head movement and skin conductance via a multisystem physiological data monitoring system (Nexus-X, Mindmedia, The Netherlands).

### Analysis of stride interval time series

The mean, standard deviation, coefficient of variation (which is the ratio of the standard deviation to the mean) and the spectral exponent were estimated for stride interval time series extracted from both feet.

To determine the spectral exponents of the stride interval signals, the average wavelet coefficient (AWC) method was used. This method can be summarized as follows [46, 47]:

1. The wavelet transform of the centred version of the signal, $WV _x(\tau ,s)$, is computed using the Daubechies 12 wavelet.
2. The mean average with respect to translation coefficient $\tau$ of the magnitude of the wavelet transform is evaluated for each scale. This quantity is called:
  1 $$\begin{aligned} WV _x(s) = \left\langle |WV _x(\tau ,s)| \right\rangle _\tau \end{aligned}$$
3. The log-log plot of $WV _x(s)$ as a function of the scale *s* is plotted. The linear regression of the resulting graph is computed. The slope of this line is $H_{fBm} + 1/2$ [46].
4. The spectral exponent $\beta$ is evaluated using the expression:
  2 $$\begin{aligned} \beta = 2 H_{fBm} + 1 \end{aligned}$$

The number of scales were chosen with respect of the signal length, using the following expression:

3 $$\begin{aligned} {\text{number\;of\;scales}} = 2^n{\text{ with}}\;n \,\,= \,\, \min \left( \max \left( \left\lfloor \log _2 \left[ \frac{{\rm length} (x)}{4}\right] \right\rfloor , 7\right) , 3\right) \end{aligned}$$

### Analysis of MCA signals

#### Statistical features

In this study, four distinct statistical features where evaluated. The standard deviation, the skewness, the kurtosis along with the cross-correlation coefficient of right and left MCA signals were extracted and compared. The first three parameters characterize the shape of the signal’s distribution [48], while the last one characterizes the similarity between two signals.

The kurtosis of a distribution can be expressed as [48]:

4 $$\begin{aligned} \beta _2 = \dfrac{\dfrac{1}{n} \displaystyle \sum _{i = 1}^{n} (x_i - \mu )^4}{\left[ \dfrac{1}{n} \displaystyle \sum _{i = 1}^{n} (x_i - \mu )^2 \right] ^2} \end{aligned}$$

where $\mu$ is the mean of the signal and *n* is its length.

The expression of the skewness is [48]:

5 $$\begin{aligned} \beta _1 = \dfrac{\dfrac{1}{n} \displaystyle \sum _{i = 1}^{n} (x_i - \mu )^3}{\sqrt{\left[ \dfrac{1}{n} \displaystyle \sum _{i = 1}^{n} (x_i - \mu )^2 \right] ^3}} \end{aligned}$$

In this study, similarity between right MCA and left MCA signals was calculated as follows [49]:

6 $$\begin{aligned} CC _{X|Y}=\frac{1}{n}\sum _{i=1}^n x_i y_i \end{aligned}$$

where *X* and *Y* represent signals from the right and the left side of the MCA.

#### Information-theoretic features

With regard to the information-theoretic feature space, the Lempel–Ziv complexity (LZC), which represents the amount of new pattern formation in finite time sequences built upon the original signal (which can be interpreted as the randomness, the predictability and the regularity of a given discrete-time signal) [50] and the entropy rate, which represents the statistic degree of recurrence of patterns in a stochastic process [51].

In order to obtain a finite time sequence to compute the LZC, the signal is divided into $\alpha$ finite equal spaces using $\alpha -1$ threshold values given as $T_h | h \in \{1, \ldots , \alpha - 1\}$ [52].

Then, portions of the quantized signal $X_1^n=\{x_1,x_2,\ldots ,x_n\}$ are assembled to shape blocks such that [53]:

7 $$\begin{aligned} X_j^\ell =\{x_j,x_{j+1},\ldots ,x_\ell \}, 1 \le j \le \ell \le n \, | \, j , \quad \ell \in \mathbb {Z}^+ \end{aligned}$$

where the length of each block is given as $L = j - l + 1$, and the length of the signal is *n*. The blocks are a time series of successive data.

Then, for each length *L*, an analyse of each block is performed the following way: a counter *c*(*j*) is defined to illustrate the amount of new pattern formation. If the sequence represented in a block has not appeared in a previous analysis, this counter is incremented by one.

Eventually, the LZC is computed as given herein bellow:

8 $$\begin{aligned} {\rm LZC} = \frac{c(n) \log _{\alpha }(n)}{n} \end{aligned}$$

where *c*(*n*) is the value that the counter takes when the entire analysis is performed and $\alpha$ represents the total number of quantized levels in the signal, chosen such as $\alpha = 100$ in that study.

To compute the entropy rate $\rho$ of a given stochastic process, the pattern distribution is first normalized to feature zeros mean and unit variance. This distribution is then quantized using 10 equally divided discrete amplitude levels, taking integer values between 0 and 9. The quantized distribution $\tilde{X} = \{\tilde{x}_1, \tilde{x}_2,\ldots ,\tilde{x}_n\}$ is then decomposed into blocks comprised of successive samples, with length *L* such that $10 \le L \le 30, \; L \in \mathbb {Z}^+$. Subsequently, the distribution made up of the different blocks was encoded into $\Omega _L=\{ \omega _1, \omega _2,\ldots ,\omega _{n-L+1}\}$ such as [54]:

9 $$\begin{aligned} \omega _i=10^{L-1} \tilde{x}_{i+L-1}+10^{L-2}\tilde{x}_{i+L-2}+ \ldots +10^0 \tilde{x}_{i} \end{aligned}$$

with $w_i$ varying from 0 to $9 \times (1 - 10^L)/(1-10) = 10^L - 1$.

The Shannon entropy $S (L)$, which represents the degree of complexity of $\Omega _L$, is defined by [54]:

10 $$\begin{aligned} S (L) = \sum _{j = 1}^{10^{L}-1} p_{\Omega _L}(j) \ln p_{\Omega _L}(j) \end{aligned}$$

where $p_{\Omega _L}(j)$ is the probability of the value *j* in $\Omega _L$, which is approximated by its sample frequency in this study.

The normalized conditional entropy is then given as [55]:

11 $$\begin{aligned} {\rm N} (L)=\frac{{\rm S} (L)-{\rm S} (L-1)+{\rm S} (1) \cdot {\rm pe} _\%(L)}{{\rm S} (1)} \end{aligned}$$

where ${\rm S} (1) \cdot {\rm pe} _\%(L)$ is a correction term defined by the multiplication of the percentage of patterns with length *L* arising only once in $\Omega _L$, ${\rm pe} _\%(L)$, with ${\rm S} (1)$ the conditional entropy estimation of the process with unit length *L*, which is the Shannon entropy of white Gaussian noise process. This term corrects the underestimation of ${\rm S} (L)-{\rm S} (L-1)$ for large lengths *L* [56]. Considering the opposite nature of the variation of the terms in the denominator (the first term decreases with *L* while the second term increases), the function ${\rm N} (L)$ exhibits a minimum $\min _{L}\left[ N(L)\right]$. This minimum is the best estimation of the normalized conditional entropy, and it can be seen as an indicator of complexity of the process. Conversely, the complement to this indicator, given as $\rho = 1 - \min _{L}\left[ N(L)\right]$, is an index of the regularity of the stochastic process, ranging between 0 and 1 [55].

For comparison between two probability density functions purposes, one can use the cross-entropy rate. This index quantifies the amount of mutual information between two given distributions, and aims to predict the data of a considered signal using the previous and current information found in another signal. The two distributions *X* and *Y* were normalized and quantized in a similar fashion than the one used to compute the entropy rate, giving as a result the two quantized distribution $\tilde{X} = \{\tilde{x}_1, \tilde{x}_2,\ldots ,\tilde{x}_n\}$ and $\tilde{Y} = \{\tilde{y}_1, \tilde{y}_2,\ldots ,\tilde{y}_n\}$.

Eventually, the cross-entropy rate $\Omega _L^{X|Y}$, which is the information amount that can be found in one of the samples of the quantized process $\tilde{X}$ when a pattern of $L-1$ samples of the quantized signal $\tilde{Y}$ is assumed, was encoded using the following code [55]:

12 $$\begin{aligned} \omega _i^{X|Y}=10^{L-1}\tilde{x}_{i+L-1}+10^{L-2}\tilde{y}_{i+L-2}+\cdots +10^0\tilde{y}_{i} \end{aligned}$$

where ${\rm S} _X(L)$, ${\rm S} _Y(L)$ and ${\rm S} _{X|Y}$ are the Shannon entropies of the distributions *X*, *Y* and $\Omega _L^{X|Y}$.

The normalized cross-entropy was then computed as:

13 $$\begin{aligned} NC _{X|Y}(L)=\frac{{\rm S} _{X|Y}(L)-{\rm S} _Y(L-1)+{\rm S} _X(1) \cdot {\rm pe} _{X|Y}(L)}{{\rm S} _X(1)} \end{aligned}$$

where ${\rm pe} _{X|Y}(L)$ is the percentage of arrangements of length *L* that were present only once in $\Omega _L^{X|Y}$ and ${\rm S} _X(1) \cdot {\rm pe} _{X|Y}(L)$ is a corrective term added for the same reasons as the one stated herein above. Similarly to the previously given algorithm, ${\rm S} _X(1)$ is the conditional entropy estimation of the stochastic process *X* for a unit length. The function ${\rm NC} _{X|Y}(L)$ features a minimum $\min _L\left[ {\rm NC} _{X|Y}(L), {\rm NC} _{Y|X}(L) \right]$. The synchronization index is then defined by $\Lambda _{X|Y}=1 -\min _L\left[ {\rm NC} _{X|Y}(L), {\rm NC} _{Y|X}(L) \right]$, and it ranges from 0 (when *X* and *Y* are independent) to 1 (when *X* and *Y* are synchronized stochastic processes).

#### Frequency features

Features were also extracted with regard to the frequency domain: the peak frequency, spectral centroid and bandwidth were computed as characterization indexes for the extracted from the TCD signals [45].

The peak frequency, given as $f_p$, is the frequency location where the largest spectral power can be found, and it is thus computed as:

14 $$\begin{aligned} f_p= \underset{f \in [{0},{f_{\max }}]} {\rm {argmax }}\left\{ | F_X(f) |^2\right\} \end{aligned}$$

where $F_X(f)$ is the Fourier transform of the signal *X* and $f_{\max }$ the spectrum’s maximum frequency, equal to the sampling frequency divided by two.

The spectral centroid, $f_c$, can be interpreted as the spectrum’s center of mass and is calculated as follows [57]:

15 $$\begin{aligned} f_c=\dfrac{\displaystyle \int _{0}^{f_{\max }} f |F_X(f)|^2 \, \mathrm df }{\displaystyle \int _{0}^{f_{\max }}|F_X(f)|^2\, \mathrm df } \end{aligned}$$

The bandwidth *BW*, representing the dispersion of the spectrum, was computed as [45]:

16 $$\begin{aligned} BW=\sqrt{\dfrac{\displaystyle \int _{0}^{f_{\max }} (f-f_c)^2|F_X(f)|^2 \, \mathrm df }{\displaystyle \int _{0}^{f_{\max }} |F_X(f)|^2 \, \mathrm df }} \end{aligned}$$

#### Time-frequency features

To extract features related to the time-scale domain, the signal was decomposed into 10 levels $W = \left\{ a_{10}, d_{10}, d_{9}, \ldots , d_{1} \right\}$ (where $a_{10}$ is the approximation coefficient and $d_{k}$ represents detail coefficient at the *k*th level [58]) using a discrete wavelet transform approach and the Meyer wavelet as a mother wavelet. The relative wavelet energy from both the approximation coefficient and detail coefficients are then computed as [59]:

17 $$\begin{aligned} \Xi _{a_{10}}=\dfrac{{\Vert a_{10}\Vert }^2}{{\Vert a_{10}\Vert }^2+\displaystyle \sum _{k=1}^{10} {\Vert d_{k}\Vert }^2 } \times 100 \end{aligned}$$

18 $$\begin{aligned} \Xi _{d_k}=\dfrac{{\Vert d_{k}\Vert }^2}{{\Vert a_{10}\Vert }^2+\displaystyle \sum _{k=1}^{10} {\Vert d_{k}\Vert }^2 } \times 100 \end{aligned}$$

where $\Vert \cdot \Vert$ is the Euclidian norm, defined as $\Vert \varvec{x} = [x_1,\ldots , x_n] \Vert = \left( {\sum x_i^2}\right) ^{1/2}$.

These metrics describe the relative energies repartition within distinct frequency bands based upon the ratio of the *k*-th level of decomposition to the total energy of the signal.

The wavelet entropy $\Omega$ is an indicator of the order of the decomposed signal [59]. It is a representation of the clustering of the wavelet energies comprised in the different decomposition levels.

19 $$\begin{aligned} \Omega =- ~ \Xi _{a_{10}} \log _2\Xi _{a_{10}} - \sum _{k=1}^{10} \Xi _{d_k} \log _2 \Xi _{d_k} \end{aligned}$$

where $\Xi _{\cdot }$ are the relative energies defined herein above.

### Statistical test

SAS version 9.3 (SAS Institute, Inc., Cary, North Carolina) was used for all statistical analyses. Our overall strategy was to keep the two trials separate and fit a sufficiently complex model in order to account for between-trial correlation to maximize the use of information and statistical power. For simpler methods such as correlation analyses, we averaged participant measures across the two trials. To examine significance of systematic differences in measures between the two trials we fitted a series of linear mixed models using the SAS MIXED procedure with each gait/blood flow measure as the dependent variable; trial as the factor of interest; and a subject random effect to account for multiple correlated measurements from the same participant. We computed baseline condition vs cognitive task difference for each measure, and fitted a similar mixed model with the said differences as dependent variables but in an intercept-only model to obtain statistical significance for the between-condition difference under two correlated trials. To examine whether participant characteristics (age, gender, height, weight, BMI), we fit another series of models with each gait/blood flow measure under each task condition as the dependent variable; each participant characteristic as the fixed effect of interest; and a participant random effect to account for multiple trials. To examine correlation coefficients across measures, we first averaged all measures across the two trials. Next, we used Pearson correlation coefficients between gait measures and blood flow measures; as well as between left and right sides for the blood flow measures. Additionally, we obtained partial correlations adjusted for participant characteristics. We are aware of the large number of measures and statistical tests performed in our analysis, and thus use false discovery rate (FDR) methodology for adjusting p-values for multiplicity [60].

## Results

The end-tidal carbon dioxide level does not influence the mean diameter of the middle cerebral arteries [61]. Consequently, it is not taken into consideration. The results are presented in tables in the form of $({\rm mean} ~\pm ~ {\rm standard deviation} )$ where the baseline period is pointed out by a “B” and the cognitive task is indicated by a “C”. R-MCA indicates the right MCA, while L-MCA indicates the left MCA. The test-retest were not found to introduce statistical differences if the FDR adjusted p-values are considered.

### Stride interval features

Feature extracted from the subjects’ stride intervals are provided in Table 1. No statistical differences between the baseline (denoted as “B”) and cognitive states (denoted as “C”) were found if the FDR adjusted p values are considered.

**Table 1:** Stride interval features

	Right foot	Left foot
Mean (s)		
B	1.24 ± 0.07	1.24 ± .07
C	1.26 ± 0.07	1.26 ± 0.07
Coefficient of variation		
B	(3.09 ± 0.97)	(2.89 ± 0.76)^a^
C	(2.76 ± 1.17)^a^	(2.44 ± 0.82)^a^
Spectral exponent $\beta$		
B	0.64 ± 0.49	0.66 ± 0.42
C	0.41 ± 0.27	0.47 ± 0.22

^a^Denotes a multiplication by $10^{-2}$

### MCA signal features

#### Time features

Table 2 summarizes time-domain feature values for the raw and the envelope signals. For every metrics considered in that table, no statistical differences between the left side and the right side were observed. However, statistical differences between cognitive state and baseline state were observed for the standard deviation of the envelope signal ($p \approx 0.018$) for both R-MCA and L-MCA, and for both the envelope and raw signals’ R-MCA and L-MCA cross-correlation ($p \approx 0.018$).

Moreover, the inspection of the mean peak blood flow velocity revealed an increase during the cognitive task ($57.8~\pm ~9.0 {\rm cm} ~s ^{-1}$ in the baseline trial vs $59.5 ~\pm ~ 10.3 {\rm cm} ~s ^{-1}$ in the cognitive trial for the R-MCA and $59.1~\pm ~8.4 {\rm cm} ~s ^{-1}$ in the baseline trial vs $59.3~\pm ~10.1 {\rm cm} ~s ^{-1}$ in the cognitive trial for the L-MCA).

Additionally, a similar increase in the standard deviation of the envelope signal (the increase if of 1.25 $cm ~s ^{-1}$ for the R-MCA and of 1.41 $cm ~s ^{-1}$ for the L-MCA) can be observed. This increase in the standard deviation of the blood flow velocity is characteristic of an increase in the blood flow: because the higher standard deviation characterizes a wider blood flow velocity dispersion, this means that there is an increase in the range covered by the minimal and maximal blood flow velocity, which is characteristic of a global increase of the blood flow velocity.

**Table 2:** Time features from raw and envelope CBFV signals

	Raw		Evvelope	
	R-MCA	L-MCA	R-MCA	L-MCA
Standard deviation				
B	0.13 ± 0.10	0.13 ± 0.08	3.77 ± 1.01^b^	4.03 ± 1.13^b^
C	0.15 ± 0.10	0.14 ± 0.08	5.02 ± 1.62^b^	5.44 ± 1.36^b^
Skewness				
B	(−4.99)^a^ ± 0.02	(−1.52)^a^ ± 0.01	−1.03 ± 0.74	−0.80 ± 0.76
C	(7.15)^a^ ± 0.02	(−0.11)^a^ ± 0.03	−1.22 ± 0.82	−1.07 ± 0.75
Kurtosis				
B	4.38 ± 2.74	5.08 ± 3.31	5.50 ± 3.95	4.73 ± 3.13
C	4.51 ± 1.67	5.11 ± 3.22	5.41 ± 2.98	4.47 ± 1.91
Cross-Correlation				
B	(7.27 ± 2.04)^ab^		0.98 ± 0.01^ab^	
C	0.02 ± 0.02^ab^		0.97 ± 0.02^ab^	

^a^Denotes a multiplication by $10^{-3}$

^b^Denotes FDR corrected statistical differences between baseline and cognitive runs

#### Information-theoretic features

Information-theoretic features from both CBFV raw and envelope signals are presented in Table 3. The LZC and synchronization index of the envelope signals exhibited significant statistical differences between the two states ($p < 0.02$).

**Table 3:** Information-theoretic features from raw and envelope CBFV signals

	Raw		Envelope	
	R-MCA	L-MCA	R-MCA	L-MCA
LZC				
B	0.71 ± 0.04	0.71 ± 0.04	0.70 ± 0.02^b^	0.70 ± 0.02^b^
C	0.70 ± 0.04	0.70 ± 0.03	0.68 ± 0.02^b^	0.68 ± 0.03^b^
Entropy rate				
B	0.09 ± 0.10	0.10 ± 0.15	(7.61 ± 1.75)^a^	(7.56 ± 2.43)^a^
C	0.11 ± 0.13	0.11 ± 0.11	0.01 ± 0.01	0.01 ± 0.01
Synchronization index				
B	0.17 ± 0.13		0.07 ± 0.03^b^	
C	0.16 ± 0.12		0.12 ± 0.06^b^	

^a^Denotes a multiplication by $10^{-3}$

^b^Denotes FDR corrected statistical differences between baseline and cognitive runs

#### Frequency features

Table 4 summarizes the frequency characteristics of raw and envelope signals. These metrics do not display any significant statistical differences between the left and right MCA raw and envelope signals. The spectral centroid of the R-MCA raw signal was found to feature statistical differences ($p = 0.038$), while the bandwidth of the raw CBFV shows statistical differences for the L-MCA, with $p < 0.01$.

**Table 4:** Frequency features from raw and envelope CBFV signals (values are in Hertz)

	Raw		Envelope	
	R-MCA	L-MCA	R-MCA	L-MCA
Spectral centroid				
B	546 ± 47.3^a^	523 ± 62.1	2.92 ± 0.43	2.91 ± 0.40
C	504 ± 80.6^a^	488 ± 68.2	2.80 ± 0.46	2.72 ± 0.49
Peak frequency				
B	574 ± 115	539 ± 144	1.25 ± 0.62	1.08 ± 0.71
C	437 ± 202	421 ± 196	0.84 ± 0.81	0.71 ± 0.84
Bandwidth				
B	172 ± 15.8	174 ± 12.4^a^	2.38 ± 0.33	2.40 ± 0.30
C	186 ± 16.1	193 ± 17.7^a^	2.43 ± 0.24	2.37 ± 0.21

^a^Denotes FDR corrected statistical differences between baseline and cognitive runs

#### Time-frequency features

**Fig. 1:**
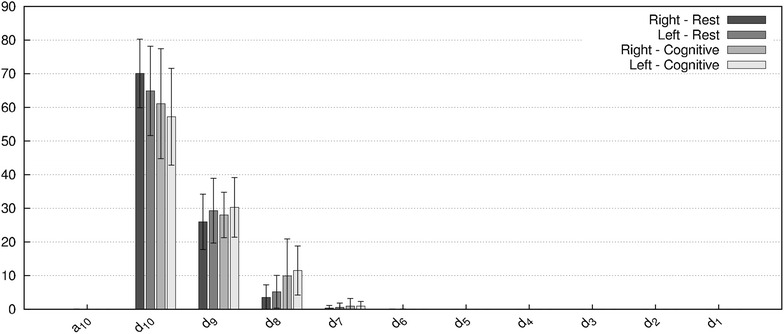
The 10 level wavelet decomposition of raw signals

Table 5 shows the wavelet entropy values for raw and envelope signals, while Figure 1 displays the wavelet decomposition of the raw CBFV signal. The envelope signal’s wavelet decomposition is not displayed because almost the entire relative wavelet energy can be found in $a_{10}$ (99 %). When the wavelet entropies are considered, the R-MCA and L-MCA CBFV wavelet entropies feature statistical differences between baseline and cognitive states, with respectively $p = 5.4~\times ~10^{-3}$ and $p = 1.1~\times ~10^{-2}$.

**Table 5:** Wavelet entropy values for raw and envelope CBFV signals

	Raw		Envelope	
	R-MCA	L-MCA	R-MCA	L-MCA
$$\Omega$$				
B	1.02 ± 0.21^ a^	1.11 ± 0.27^ a^	0.03 ± 0.02	0.04 ± 0.03
C	1.22 ± 0.23^ a^	1.32 ± 0.23^ a^	0.05 ± 0.06	0.06 ± 0.03

^ a^Denotes FDR corrected statistical differences between baseline and cognitive runs

Figure 1 exhibits that the majority of wavelet energy is massed in high order detail coefficients (mainly for $7 \le k \le 10,~k \in \mathbb {Z}^+$). The coefficient $d_{10}$ accounts for the highest percentage of wavelet energy (between 70 % for the R-MCA in the baseline state and 58 % for the L-MCA in the cognitive state). The energy contained is the $d_{10}$ band is higher in the baseline state than in the cognitive state. A similar observation can be drawn with energy repartition in the $d_{10}$ of the R-MCA being higher than the energy of the L-MCA wavelet decomposition. The $d_9$ band behaves differently, and no particular trend emerges in this detail band. The behavior of the $d_8$ band is the inverse of the $d_{10}$ band: indeed, the energy proportion of the $d_8$ band increases from the baseline state to the cognitive state and from the R-MCA to the L-MCA. Statistical differences between the baseline and cognitive state can be found in the bands $d_{10}$ and $d_8$ for the raw R-MCA and L-MCA signals ($p~\le ~0.03$).

#### Correlation between left and right MCA blood flow

Statistical significance for the correlation between the left and right MCA CBFV are only found for the wavelet decomposition of the raw CBFV signals: the levels $d_5$ to $d_7$ exhibit a correlation coefficient greater than 0.8 with $p \le 0.005$. The levels $d_2$ and $d_1$ feature a correlation coefficient greater than 0.9 with $p \ll 0.001$.

### Impact of anthropometric variables on features

The anthropometric variables were not found to have an impact on any of the features.

### Correlation between gait and MCA features

The Pearson correlation coefficient $\rho$ were thresholded using the following rule:

20 $$\begin{aligned} \rho _{th} = \left\{ \begin{array}{ll} 1\quad{\rm if} |\rho | > 0.7 \, {\rm and} \, p < 0.05 \\ 0\quad{\rm otherwise} \, \end{array} \right. \end{aligned}$$

**Fig. 2:**
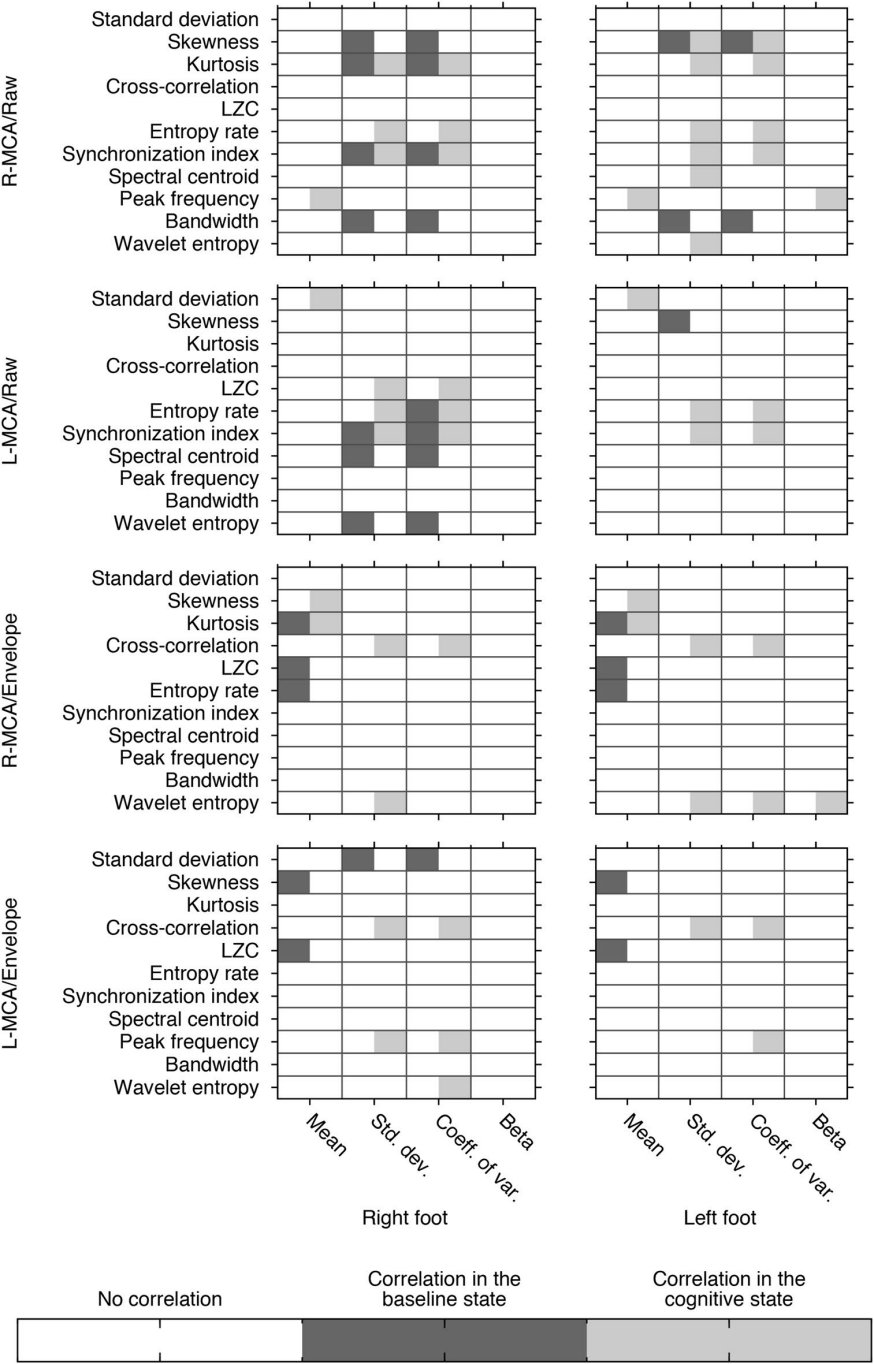
Anthropometric-variable adjusted correlation between the *left* and *right* foot gait features and the *left* and *right* MCA blood flow velocity features

Figure 2 displays the correlation of the gait features and the CBFV features. A dark-gray rectangle represents an absolute correlation of at least 0.7 in the baseline state, while a light-gray rectangle represents a correlation in the cognitive state. Some features exhibit correlations in both states (e.g. L-MCA raw signal synchronization index is always correlated to the right foot standard deviation or R-MCA raw signal kurtosis is correlated to both the right foot standard deviation and coefficient of variation), while some features’ correlation changes from one state to another (e.g. the correlation between the R-MCA Lempel–Zif complexity and the right foot mean stride interval observed in the baseline state is not present in the cognitive state, or the correlation between the left foot standard deviation and the entropy rate of the L-MCA raw signal observed in the cognitive state is not present in the baseline state). From this figure, it is clear that with the cognitive processing added task there was a greater number of correlated gait and blood flow velocity features: in the baseline test, 9.1 % of the potential relations between gait and cerebral blood flow were correlated, while in the cognitive processing added task condition, 14.5 % of the potential gait and blood flow measures were related.

More specifically, all the stride-related features except the right foot spectral exponent coefficient are found to be correlated with at least one feature extracted from the cerebral blood flow signals. Indeed, four blood flow related features are found to be correlated to both the left and right feet mean stride interval (peak frequency of the R-MCA raw signal, standard deviation of the L-MCA raw signal, both the skewness and kurtosis of the R-MCA envelope signals). The standard deviation and coefficient of variation of the right foot stride interval both are correlated with 10 of the features of the CBFV (with 9 of the correlated being the same for the left and right foot). The same stride related features when the left foot is considered are correlated with 11 and 10 features of the CBFV, respectively. Lastly, the spectral exponent coefficient for the left foot stride interval is correlated to two of the central blood flow velocity signals features: the R-MCA raw signal peak frequency and the R-MCA envelope signal wavelet entropy.

## Discussion

Our major finding is that there are statistical differences in cerebral blood flow velocities in MCA between the baseline and cognitive states during walking. These differences were observed both in the raw signals and in the envelope signals (i.e., the maximal velocities) associated with cerebral blood flow velocities.

The statistical differences revealed in this study between blood flow during walking and blood flow during walking with an added cognitive processing task illustrate the suggested performance pattern seen in older adults with greater difficulty walking under challenging conditions such as walking and talking or walking and thinking. Under the challenge of the added cognitive task, it was more difficult to maintain usual gait performance. The difficulty manifested in gait as largely greater inconsistency in the pattern of strides than in the mean of the stride interval time series. This difficulty was also mirrored in the cerebral blood flow. Thus, under the cognitive processing task condition, the range of gait performance was expanded for some variables, despite no change in the group mean for the gait characteristics. The expanded range of gait performance may underlie the different and greater number of correlations between gait and blood flow in the walking plus cognitive processing condition. A cognitively challenging task during walking may alter the range of gait performance, which is also associated with greater cerebral blood flow or ‘work’ of the brain.

Among the stride measures, the standard deviation of the stride interval exhibited the strongest correlation with blood flow velocity features. This finding may indicate that while the cognitive processing did not disrupt gait described by the mean of the stride interval, the added cognition may have influenced the consistency of gait performance (eg, standard deviation). While all participants accomplished the task of walking and thinking on the treadmill with no change in the mean stride characteristics, the difference in the level of cognitive challenge for participants was apparent in the inconsistency of gait. Greater cognitive challenge for some was associated with greater blood flow. Moreover, the increase in the bandwidth of the raw signals denotes a change in individual behavior of the erythrocytes, as a higher bandwidth value denotes higher spectral spread, which is caused by more erratic flow of red blood cells. The lack of statistical differences between the first and second trials denotes that the extracted features tend to be trial-independent.

## Limitations of the study

A potential limitation of this study comes from the fact that the participants were instructed to walk on a treadmill: indeed, changes in the gait characteristics are very likely concealed by the walking rhythm induced by the treadmill.

## Conclusion

In this paper, the dual task methodology exhibited that the cerebral blood flow velocity signals in the baseline and cognitive states during walking are statistically different. These differences can be found in extracted features for both the raw and envelope of the L-MCA and R-MCA central blood flow velocity signals. While the changes in gait features were subtle, the different and more relations between gait and blood flow with the added cognitive processing during walking suggests cerebral blood flow velocity may represent the work of the brain when thinking and walking.
